# Linear and Non-linear Quantification of the Respiratory Sinus Arrhythmia Using Support Vector Machines

**DOI:** 10.3389/fphys.2021.623781

**Published:** 2021-02-05

**Authors:** John Morales, Pascal Borzée, Dries Testelmans, Bertien Buyse, Sabine Van Huffel, Carolina Varon

**Affiliations:** ^1^STADIUS Center for Dynamical Systems, Signal Processing and Data Analytics, Department of Electrical Engineering (ESAT), KU Leuven, Leuven, Belgium; ^2^Leuven.AI - KU Leuven Institute for AI, KU Leuven, Leuven, Belgium; ^3^Department of Pneumology, UZ Leuven, Leuven, Belgium; ^4^e-Media Research Lab, Department of Electrical Engineering, KU Leuven, Leuven, Belgium

**Keywords:** respiratory sinus arrhythmia, heart rate variability, support vector machines, nonlinear methods, biomedical data processing, electrocardiogram, cardiorespiratory interactions

## Abstract

Respiratory sinus arrhythmia (RSA) is a form of cardiorespiratory coupling. It is observed as changes in the heart rate in synchrony with the respiration. RSA has been hypothesized to be due to a combination of linear and nonlinear effects. The quantification of the latter, in turn, has been suggested as a biomarker to improve the assessment of several conditions and diseases. In this study, a framework to quantify RSA using support vector machines is presented. The methods are based on multivariate autoregressive models, in which the present samples of the heart rate variability are predicted as combinations of past samples of the respiration. The selection and tuning of a kernel in these models allows to solve the regression problem taking into account only the linear components, or both the linear and the nonlinear ones. The methods are tested in simulated data as well as in a dataset of polysomnographic studies taken from 110 obstructive sleep apnea patients. In the simulation, the methods were able to capture the nonlinear components when a weak cardiorespiratory coupling occurs. When the coupling increases, the nonlinear part of the coupling is not detected and the interaction is found to be of linear nature. The trends observed in the application in real data show that, in the studied dataset, the proposed methods captured a more prominent linear interaction than the nonlinear one.

## 1. Introduction

In the context of network physiology, three independent forms of cardiorespiratory coupling have been described, namely, cardiorespiratory phase synchronization, time delay stability, and respiratory sinus arrhythmia (RSA). These have been demonstrated to be independent and to have effects in different time scales. Furthermore, biomarkers to quantify these interactions have been shown to be better to evaluate certain conditions and diseases compared to the analysis of the cardiac and respiratory systems individually (Bartsch and Ivanov, 2014). RSA is the most studied one and it is the main focus of this paper. It is observed as changes in heart rate (HR) in synchrony with the respiratory cycle. During inhalation the HR accelerates and during exhalation it decelerates. Despite the fact that RSA was already described in 1733 (Billman, 2011), the mechanisms producing it and its function are not yet fully understood. However, RSA has been suggested as a biomarker for illnesses and conditions such as diabetes (Mackay, 1983), aging (Hrushesky et al., 1984), sleep apnea (Bonsignore et al., 1995), heart failure (Peltola et al., 2008), anxiety disorders (Gorka et al., 2013), and stress (Varon et al., 2018).

The non-invasive evaluation of the RSA can be done using the tachogram (i.e., time intervals between consecutive R-peaks) as a heart rate variability (HRV) representation (Sörnmo and Laguna, 2005). The power spectral density (PSD) estimation of the tachogram is used to derive indices of HRV in the frequency domain (Berry et al., 2012). Here, the level of activity of the sympathetic and parasympathetic branches of the autonomic nervous (ANS) system can be assessed by analyzing different frequency power bands. The low frequency (LF: 0.04–0.15) band has been hypothesized to contain information of both, sympathetic and parasympathetic modulators. The high frequency (HF: 0.15–0.4 Hz) band is widely accepted to reflect the parasympathetic modulation and the action of the respiration (Akselrod et al., 1981; Camm et al., 1996). However, this interpretation of the HF might result in misleading interpretations, in particular when the respiratory rate appears outside the HF (Brown et al., 1993; Schipke et al., 1999; O'Callaghan et al., 2015; Shader et al., 2018). If the respiratory rate is higher than the upper limit of the HF, such as during exercise, the parasympathetic activation is underestimated. Furthermore, during activities in which a slower breathing rate is observed, such as during relaxation, the physiological interpretation of the power bands in the PSD of the HRV according to the standard can be misleading because the respiratory rate goes below the HF band. As a result, the sympathetic activation is overestimated and the vagal component is underestimated (Camm et al., 1996).

To overcome this limitation, the unconstrained methodology to assess the ANS, described in Varon et al. (2018), can be used. With this method, the HRV is decomposed into a component linearly correlated with respiration, and a residual one that captures possibly nonlinear respiratory influences as well as the action of HRV modulators different from respiration. Even though this method has been shown to better quantify the RSA as well as the sympathetic and parasympathetic activity during different conditions, it is not able to separate the possible nonlinear respiratory influences of the respiration in the HRV.

The analysis of these nonlinear components has been shown to be important for some applications. For instance, the work in Loula et al. (1994), presents an interpretation of the non linear part of the RSA during anesthesia, finding differences between measurements taken during baseline and propofol administration. This work was then extended in Chen et al. (2009), where the non linearities of the cardiorespiratory coupling were analyzed for different propofol doses. The latter paper found that the nonlinear part of the RSA remains constant at different drug levels. Another example is the work presented by Caicedo et al. (2014) which shows that a quantification of the nonlinear respiratory influence in HRV using Kernel principal component regression improved the performance of the classification of apnea events compared to a pure linear model. A last example is the work shown in Yeh et al. (2019) where an important contribution of RSA in the fractal properties of HRV is evidenced. This was then applied to improve the assessment of patients with congestive heart failure. These applications suggest that a framework to evaluate the linear and nonlinear components of the RSA would be useful.

To answer to this need, the unconstrained estimator described in Varon et al. (2015b) was extended in Varon et al. (2019), where a method based on least-squares support vector machines was proposed to extract the linear and nonlinear components of the cardiorespiratory interactions from a dataset recorded during autonomic blockade. Results suggested that the nonlinear interactions are mediated by different control mechanisms. In addition, the quantification of the linear part of the interaction is shown to underestimate the RSA due to the suppression of the nonlinear component.

In Varon et al. (2019), the coupling was described for a specific dataset of autonomic blockade with a limited number of subjects. The current paper complements this work presenting a method to quantify RSA based on support vector machines (SVM). It allows to analyze the linear and non linear contributions of the respiratory influences in the HRV representations. These methods are applied in simulated data in which the strength of the linear and nonlinear components of the coupling are controlled. Furthermore, the methods are used to analyze the change of coupling during sleep stages in a dataset of sleep apnea patients. The paper is organized as follows: section 2 describes the datasets and methods. Section 3 shows the results and discusses them. Finally, section 4 presents the conclusions and future directions.

## 2. Materials and Methods

### 2.1. Simulation

A simulation model is used in this paper to evaluate the proposed methodology for the estimation of signal interactions. The goal is to understand the way in which the proposed parameters quantify the interaction between two systems when linear and nonlinear components are present. It uses the model given by the following equations (Papana et al., 2013):

(1) $$\begin{array}{l} x_{1}\left(n\right)=1.2x_{1}\left(n-1\right)-0.7x_{1}\left(n-2\right)+0.1N\left(\sigma ,\mu \right) \end{array}$$

(2) $$\begin{array}{l} x_{2}\left(n\right)=0.5x_{2}\left(n-1\right)-C_{1}x_{1}\left(n-1\right)-C_{2}x_{1}^{2}\left(n-1\right)\\ \begin{array}{l} \;+0.1N\left(\sigma ,\mu \right), \end{array} \end{array}$$

with N a Gaussian noise with zero mean and unitary standard deviation. Here, an interaction between *x*_1_ and *x*_2_ is simulated. It consist of a linear and a nonlinear component. The strength of the linear part is defined by the coefficient *C*_1_ and the strength of the nonlinear part by the coefficient *C*_2_. Two scenarios are tested. In the first one, the coefficient *C*_2_ is set to zero to consider only linear interactions. In the second, the nonlinear effect is included using *C*_2_ = 2−*C*_1_. This bounding to the value of *C*_2_ was imposed to always have linear and nonlinear interactions, and being able to control the weight of one component compared to the other. For both scenarios, 20 realizations of signals are generated while varying *C*_1_ in the interval [0 1.8], in steps of 0.2.

### 2.2. Real Data

**Figure 1:**
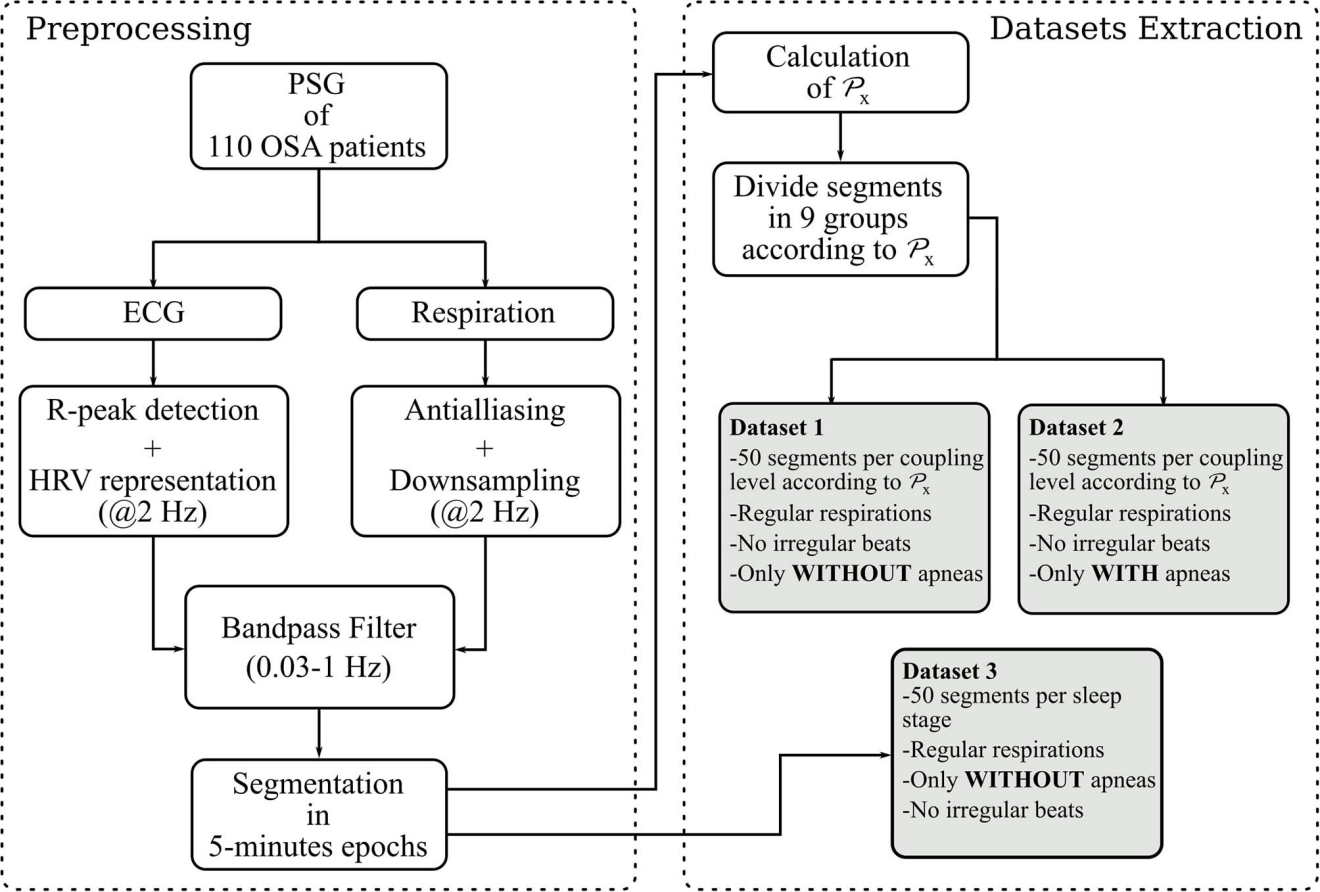
Steps followed to built the datasets. The parameter $P_{x}$ is a state-of the-art quantification of the RSA used in this paper as reference. It quantifies the proportion of power in the HRV linearly correlated with the respiration.

The procedure to preprocess the data and extract the segments used to calculate and evaluate the RSA estimates is illustrated in Figure 1.

#### 2.2.1. Reference RSA Estimation

A state-of-the-art RSA estimate is used as gold standard to built a dataset of HRV and respiratory segments with known linear coupling level. It is based on orthogonal subspace projections (Varon et al., 2018) and, to compute it, two vectors ***x*** and ***y*** containing the samples of the respiration and HRV respectively, are defined. These are used to decompose ***y*** into one component linearly correlated to ***x*** and a second one with residual information. To this end, a time-delay embedding of ***x*** is constructed to generate a subspace ***Q***. Afterwards, ***Q*** is used to calculate a projection matrix ***P***, given by,

(3) $$\begin{array}{l} P=Q{\left(Q^{T}Q\right)}^{-1}Q^{T}. \end{array}$$

With this, the component in the HRV linearly correlated with the respiration is derived as,

(4) $$\begin{array}{l} y_{r}=Py. \end{array}$$

***y***_*r*_ allows to calculate the percentage of variance relative of the linear respiratory influences on the HRV with respect to the total HRV variance as,

(5) $$\begin{array}{l} P_{x}=\frac{{y_{r}}^{T}y_{r}}{y^{T}y}. \end{array}$$

#### 2.2.2. Data and Preprocessing

The datasets analyzed in this paper were derived from 110 Polysomnography recordings of OSA patients with different severities of OSA and associated comorbidities. The recording of this dataset and its inclusion in this study was approved by the ethical committee of the university hospital UZ Leuven (S53746, S60319). More details about the recordings are given in Deviaene et al. (2020). Sleep specialists provided annotations of apneas and sleep stages. The OSA severity was assessed with the Apnea Hypopnea Index (AHI), i.e., average number of apneic events per hour of sleep. The apneas were annotated according to the AASM 2012 scoring rules (Berry et al., 2012). The demographics are shown in Table 1. The ECG and thoracic respiratory inductive plethysmograph signals were acquired with a sampling frequency of 500 Hz. The R-peaks in the ECG were detected using the approach described in Varon et al. (2015b). Afterwards, these were used to calculate the RR interval time series, which were then interpolated to a sampling frequency of 2 Hz, and used as the HRV representation. The respiratory signals were downsampled to 2 Hz after applying an antialiazing filter. Both, HRV and respiration, were then filtered to preserve only frequency components between 0.03 and 1 Hz with a 4th order butterworth filter. This filter was applied in forward and backward directions to avoid phase distortion. Next, the respiratory and HRV signals were segmented into 5-min epochs. In addition, the power spectral density (PSD) estimation of the respirations on each segment was derived using the Welch algorithm with a hamming window of 40 and 20 s overlap.

**Table 1:** Demographic information.

***N***	**Age**	**BMI**	**AHI**	**Sex**
	**Years**	**Kg/m^**2**^**	**Events/h**	
110	47.3 ± 10.6	29.3 ± 4.6	37.8 ± 23.8	M: 82
	(38–55)	(25.9–32.8)	(21.4–53.25)	W: 28

*The age, BMI, and AHI are given as the mean values ± the standard deviation*.

*Below are the ranges given as (25th percentile–75th percentile)*.

#### 2.2.3. Derivation of the Datasets

**Figure 2:**
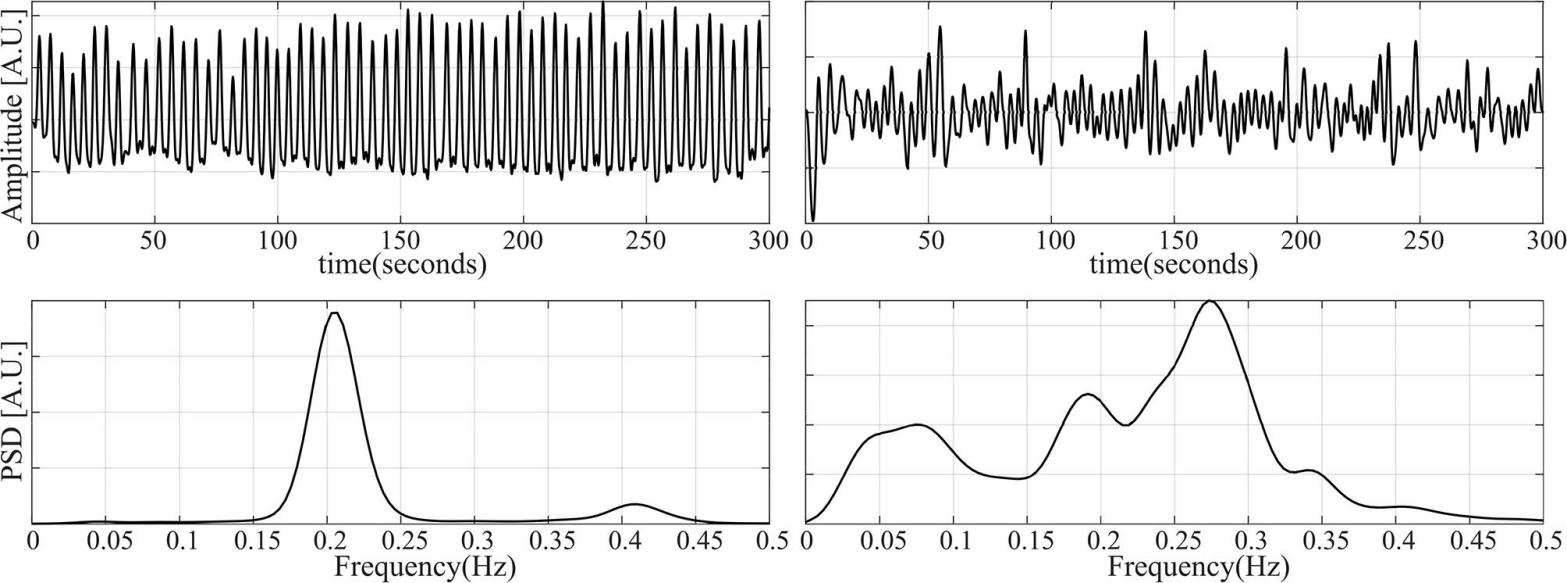
Examples of some of the respiratory segments used in this study. **(Left)** A case of an epoch free of apnea. **(Right)** An epoch during an apneic event.

With the aforementioned segments, three datasets are constructed. For the first one, the cardiorespiratory coupling is estimated using $P_{x}$. The epochs are then grouped by their $P_{x}$ level in 9 bins of 0.1, ranging from 0 to 0.9. Next, 50 randomly selected epochs per bin are visually chosen ensuring that they do not contain artifacts, irregular beats nor apneas. In addition, respiratory signals with an irregular pattern are discarded by visual inspection of the PSD. The second dataset is made following the same steps, but only segments containing apneas are included. Figure 2 illustrates examples of typical respiratory segments included in the datasets with their PSD estimation. In the third dataset, 50 randomly selected clean segments per sleep stage are chosen using the annotations given by the sleep specialists. For some groups, there are <50 segments meeting the conditions to be included. The distribution of the epochs is summarized in Table 2.

**Table 2:** Distribution of the segments per dataset.

**Dataset**	**Group**	**# Segments**	**# Subjects**	**AHI**	**Age**
Dataset 1	0.0–0.1	50	27	33 ± 16	53 ± 10
	0.1–0.2	50	32	29 ± 13	51 ± 10
	0.2–0.3	50	36	33 ± 16	46 ± 11
	0.3–0.4	50	33	34 ± 17	48 ± 12
	0.4–0.5	50	33	37 ± 19	45 ± 11
	0.5–0.6	50	30	37 ± 19	45 ± 11
	0.6–0.7	50	30	39 ± 19	43 ± 10
	0.7–0.8	50	18	38 ± 18	40 ± 10
	0.8–0.9	17	7	41 ± 18	39 ± 9
Dataset 2	0.0–0.1	50	27	38 ± 16	53 ± 10
	0.1–0.2	50	38	39 ± 19	51 ± 11
	0.2–0.3	50	39	43 ± 22	48 ± 12
	0.3–0.4	50	38	42 ± 21	47 ± 11
	0.4–0.5	50	38	45 ± 22	45 ± 10
	0.5–0.6	50	31	55 ± 22	44 ± 10
	0.6–0.7	50	30	49 ± 21	45 ± 12
	0.7–0.8	19	12	49 ± 28	44 ± 12
	0.8–0.9	3	3	42 ± 20	51 ± 10
Dataset 3	Wake	50	33	30 ± 14	50 ± 9
	REM	50	32	28 ± 13	45 ± 11
	NREM 1	34	24	33 ± 16	53 ± 9
	NREM 2	50	33	29 ± 12	46 ± 12
	NREM 3	50	37	33 ± 15	44 ± 11

### 2.3. Quantification of the Cardiorespiratory Coupling

In this paper, the hypothesis that the linear and nonlinear components of the RSA are the result of different mechanisms is tested. To this end, a method based on multivariate autoregressive models built with support vector machines (SVM) is used. The goal is to predict the present samples of the HRV using the past information in the respiration. The change of the proportion of variance captured by the prediction resulting from modifying the kernel of the model might reflect the type of relationship between the cardiac and respiratory systems^1^.

#### 2.3.1. SVM for Function Estimation

To build the SVM regression model, the samples in the HRV are estimated using the past respiratory information. Given are $x_{n}^{-}$ ∈ *IR*^*L*^, a vector of *L* past samples of the respiration, and *y*_*n*_ the corresponding present sample of the HRV signal, with *L* the model order. The definition of L will be described in section 2.3.2.

Given a training set ${\left\{x_{n}^{-},y_{n}\right\}}_{n=1}^{N}$, the following regression problem in the primal space can be formulated using the SVM framework as,

(6) $$\begin{array}{l} \underset{w,b,\xi ,\xi^{*}}{\text{min}}J_{P}\left(w,\xi ,\xi^{*}\right)=\frac{1}{2}w^{T}w+c\frac{1}{2}\overset{N}{\underset{n=1}{\sum }}\left(\xi_{n}+\xi_{n}^{*}\right)\\ \text{ such that }y_{n}-w^{T}\varphi \left(x_{n}^{-}\right)-b\leq \epsilon +\xi_{n},\;n=1,...,N\\ {\;w}^{T}\varphi \left(x_{n}^{-}\right)+b-y_{n}\leq \epsilon +\xi_{n}^{*},\;n=1,...,N\\ {\;\xi }_{n},\;\xi_{n}^{*}\geq 0,\;n=1,...,N, \end{array}$$

where ***w*** is a vector of weights, $\varphi \left(.\right):IR^{L}\to IR^{L_{h}}$ is a function that maps $x_{n}^{-}$ into a higher dimensional feature space of dimension *L*_*h*_, ξ_*k*_ as well as $\xi_{k}^{*}$ are slack variables, *b* is a bias term, *c* is a regularization term determining the tolerance to regression errors, and ϵ is the required accuracy for the solution of the problem. In order to solve these equations, the Lagrangian and the conditions for optimality are applied to formulate the following dual problem,

(7) $$\begin{array}{l} \underset{\alpha ,\alpha^{*}}{\text{min}}J_{D}\left(\alpha ,\alpha^{*}\right)=-\frac{1}{2}\overset{N}{\underset{n,m=1}{\sum }}\left(\alpha_{n}-\alpha_{n}^{*}\right)\left(\alpha_{m}-\alpha_{m}^{*}\right)K\left(x_{n}^{-},x_{m}^{-}\right)\\ \;-\epsilon \overset{N}{\underset{n=1}{\sum }}\left(\alpha_{n}+\alpha_{n}^{*}\right)+\overset{N}{\underset{n=1}{\sum }}y_{n}\left(\alpha_{n}-\alpha_{n}^{*}\right)\\ \text{ such that }\overset{N}{\underset{n=1}{\sum }}\left(\alpha_{n}-\alpha_{n}^{*}\right)=0\\ \;\alpha ,\alpha_{n}^{*}\in \left[0,c\right], \end{array}$$

where $K\left(x_{n}^{-},x_{m}^{-}\right)=\varphi {\left(x_{n}\right)}^{T}\varphi \left(x_{m}\right)$, is the kernel function and the α's correspond to the Lagrange multipliers, with α > 0 for the support vectors, and α = 0 otherwise. These can be interpreted as weights applied to the samples used to train the model. Finally, the solution to (6) becomes,

(8) $$\begin{array}{l} y_{n}\left(x^{-}\right)=\overset{N}{\underset{n=1}{\sum }}\left(\alpha_{n}-\alpha_{n}^{*}\right)K\left(x^{-},x_{n}^{-}\right)+b. \end{array}$$

It is hypothesized that the estimation of *y*_*n*_ using $x_{n}^{-}$ is better if the coupling between the two signals is stronger.

The selection of the kernel function, determines if the regression problem is solved considering only the linearities or both, the linearities and nonlinearities. For this, two kernels are used. The first one is the linear kernel, defined as,

(9) $$\begin{array}{l} K\left(x^{-},x_{n}^{-}\right)=x_{n}^{-T}x^{-}. \end{array}$$

The second one is the radial basis function (RBF) kernel, defined as,

(10) $$\begin{array}{l} K(x^{-},x_{n}^{-})=e^{\left(-\|x^{-}-x_{n}^{-}{\|}_{2}^{2}/\sigma^{2}\right)},\text{ with }\sigma^{2}\text{ the kernel bandwidth}.. \end{array}$$

As a result of the application of the RBF kernel, the regression problem is solved taking into account the linear as well as the nonlinear relationship between the signals.

It is important to mention that, to build the regression models, it is necessary to tune some parameters. The kernel bandwidth, σ^2^, was tunned using the value that maximized the Shannon entropy of the kernel matrix (Varon et al., 2015a). The regularization term, *c* of Equation (6), was given by the interquartile range of the HRV divided by 1.349. This calculation is a robust measure of scale, that quantifies the standard deviation of the response variables. The accuracy parameter, ϵ of Equation (6), was set to *c*/10. This selection of the parameters is a rule of thumb used in previous works (Ruta et al., 2019), and resulted in more consistent results over different executions than other tuning approaches.

After training the regression model, this is used to make two predictions, namely *y*_*l*_ and *y*_*k*_, using a different Kernel each time. With these, two parameters are calculated:

(11) $$\begin{array}{l} P_{x}^{l}=\frac{y_{l}^{T}y_{l}}{y^{T}y}\text{ and }P_{x}^{k}=\frac{y_{k}^{T}y_{k}}{y^{T}y}. \end{array}$$

The hypothesis here is that $P_{x}^{l}$ quantifies the percentage of variance in the HRV linearly explained by the respiration and $P_{x}^{k}$ captures the portion of the variance in the HRV explained by both the linear and possibly nonlinear interaction with the respiration.

#### 2.3.2. Model Order Selection

The selection of the parameter *L* is important because it defines the number of past samples in the respiration considered to be relevant to predict the HRV. For this reason, *L* determines the dynamics that can be captured by the regression model. Methods exist to select this parameter. Two of them are the Akaike's information criterion and the minimum description length. These two approaches have been found to produce inconsistent results in previous studies of the authors. More research on the best alternative to tune this value is needed and is out of the scope of the current work. For these reasons, a more empirical approach initially proposed in Morales et al. (2020), was used. To select *L*, a frequency (*F*_*r*_) representative of the respiratory dynamics is found. To this end, the frequency band in the PSD of the respiration containing the 90% of the total power is identified. Afterwards, the local maxima inside this band are found. If the number of local maxima is lower than 3, *F*_*r*_ is defined as the frequency with maximum power. In case of more than 3 maxima candidates, *F*_*r*_ is defined as the one with the lowest frequency. However, if *F*_*r*_ < 0.1 Hz, it is fixed to 0.1 Hz. The order *L* is calculated as the number of samples required to capture two periods of *F*_*r*_ (Morales et al., 2020).

### 2.4. Statistical Tests

#### 2.4.1. Analysis of Surrogates

To evaluate if the nonlinear quantifications with $P_{x}^{k}$ are significant, analysis of surrogates for multivariate data are used (Theiler et al., 1992; Schreiber and Schmitz, 2000). With this approach, pairs of surrogate segments of the HRV representations and respiratory signals are generated. The phases in the signals are randomized to eliminated the possible nonlinear interactions between them. This is done in such a way that the individual distributions are matched. In addition, the autocorrelation function of each signal, as well as the cross-correlation between the pairs are maintained.

In this paper, 24 surrogates are generated for each pair of segments. $P_{x}^{k}$ is computed in the original signals as well as in their surrogates. Then, the upper limit of the confidence interval for the mean value of the quantification in the surrogates is defined as the 95th quantile. If the parameter with the original signals is outside this upper limit, the quantification with $P_{x}^{k}$ in the segments is considered significantly different to the quantifications in the surrogates. Then, it is assumed that the time series interact in a linear and nonlinear way.

#### 2.4.2. Differences Between the Linear and Nonlinear Quantifications

First, differences between $P_{x}$ and $P_{x}^{l}$ are evaluated using the Friedman test for repeated measures. The same test is used to evaluate $P_{x}^{k}$ with respect to its linear counterparts. Second, to evaluate the existence of linear and possible nonlinear interactions in different sleep stages (in dataset 3), the Kruskall-wallis test is applied. In both cases, multiple comparisons with Bonferroni correction are done. A *p* < 0.05 was considered significant. The *p*-values are marked in the figures as follows: a *p* < 0.05 is shown with a asterisk (*), a *p* < 0.01 is marked with two asterisks (**) and a *p* < 0.001 is illustrated with three asterisks (***).

## 3. Results and Discussion

### 3.1. Simulation

**Figure 3:**
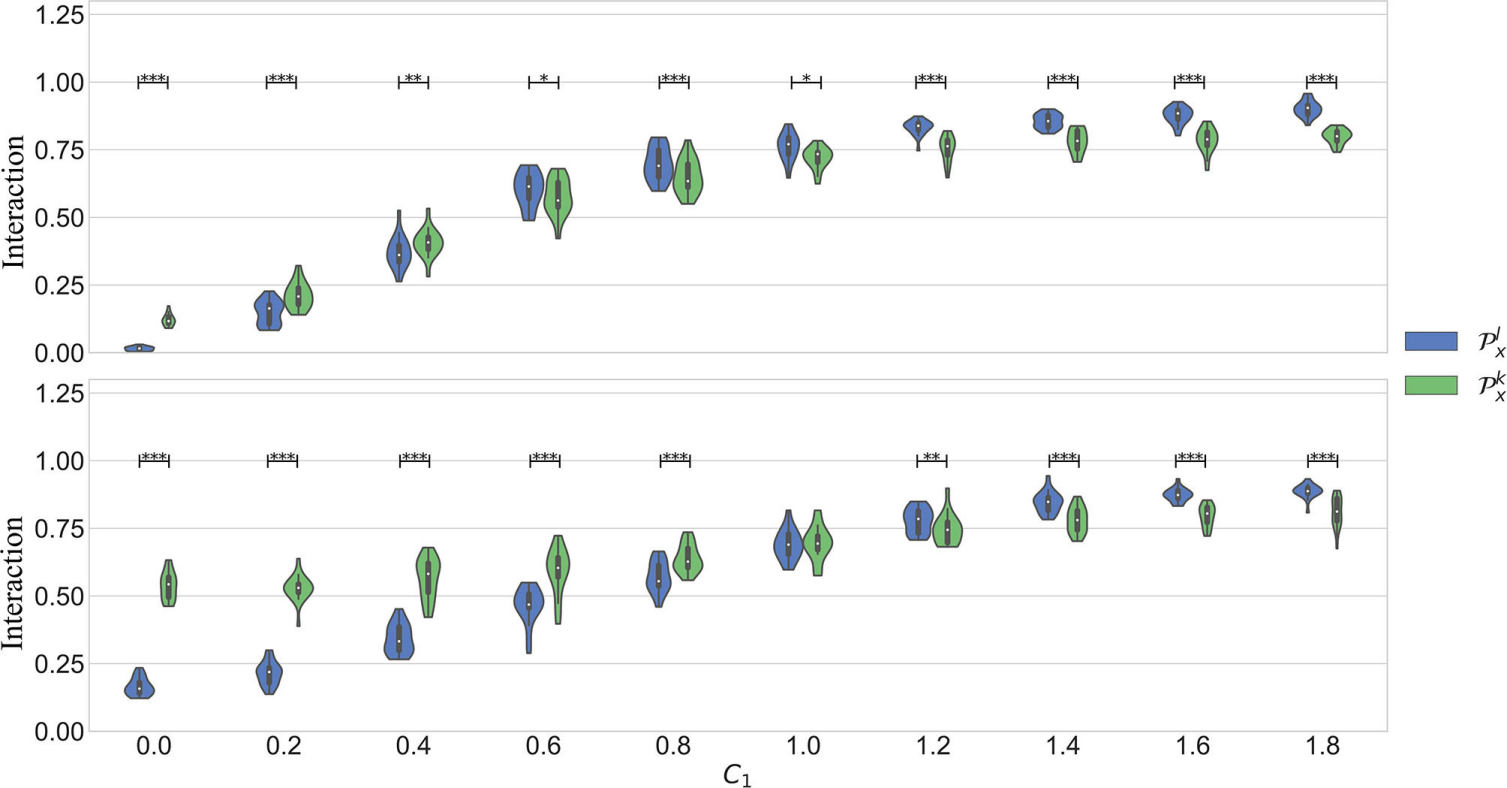
Results obtained in simulation 1. *C*_1_ models the strength of the linear part of the coupling. *C*_2_ models the nonlinear part. The figure on top shows the results in scenario 1, when *C*_2_ = 0. The bottom plot is the scenario when *C*_2_ = 2 − *C*_1_. In both cases, *C*_1_ is varied in the interval [0 1.8], in steps of 0.2.

The top plot in Figure 3 illustrates the results in the first scenario, in which only a linear part of the interaction is considered. It was expected to see values of $P_{x}^{k}$ always higher or equal than $P_{x}^{l}$. The figure shows that this is true only when the coupling is weak. However, when the coupling gets stronger, the quantification with $P_{x}^{k}$ becomes significantly lower. The analysis of surrogates confirmed that the nonlinear interactions quantified by $P_{x}^{k}$ are not significant in most of the cases.

In the second scenario, the interaction between the systems is composed of a linear and a nonlinear part. The bottom plot of Figure 3 shows the results. It is seen that $P_{x}^{k}$ is significantly higher than $P_{x}^{l}$ up to *C*_1_ = 0.8. Afterwards, when the linear component gets stronger, the linear kernel produces a significantly higher quantification. Despite that the quantification with $P_{x}^{k}$ was higher in a wider interval in this case, it is also seen that this parameter varies less with an increased linear interaction. It is well-known that the RBF kernel can act as an universal approximator. In other words, it can approximate a linear as well as a non linear type of interaction. However, the results suggest that indeed it is able to capture the more general behavior while avoiding to over fit the data. This can be seen when *C*_1_ > 1.4, when the model captures more the linear behavior.

### 3.2. Real Data

**Figure 4:**
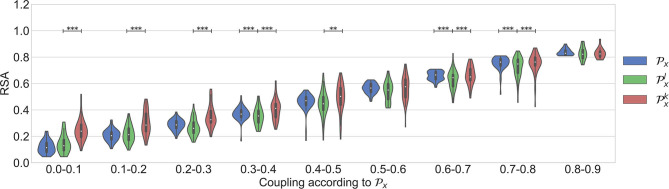
Results using the dataset of clean segments, free of apneas, and with regular respirations.

Dataset 1 includes only the clean segments without apneas and irregular heart beats. It was used to study the occurrence of nonlinear interactions when regular respiratory patterns occur. Figure 4 shows the results. As expected, an increasing trend is observed in all the parameters when the quantification of the linear coupling calculated with $P_{x}$ increases. Despite the significantly larger quantification obtained with $P_{x}^{k}$ when compared to the one with $P_{x}^{l}$, the surrogates suggested that the interactions were purely linear. Another observation is that significant differences between $P_{x}$ and $P_{x}^{l}$ were not found. This means that both parameters quantify the linear part of the cardiorespiratory interactions in a similar way. The results suggest that the linear component of the RSA is more prominent in this dataset.

**Figure 5:**
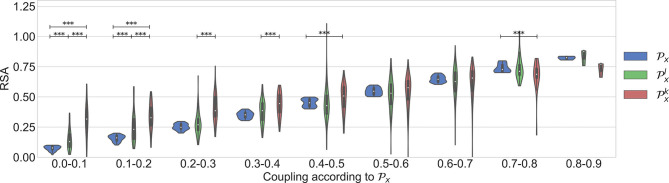
Results using the dataset of segments with apneas.

Dataset 2 was used in order to assess if respiratory signals with broader bandwidths result in a higher nonlinear component in the RSA. Figure 5 shows the results. As shown in Varon et al. (2019), in this case $P_{x}$, $P_{x}^{k}$, and $P_{x}^{l}$ are preferred to quantify the RSA than the standard HF band to avoid the effect of the broadband respiratory frequency components. As shown in the figure, significant differences between $P_{x}$ and $P_{x}^{l}$ were not found. However, it is also seen here that both parameters might quantify the cardiorespiratory coupling differently in this case, since the quantification with $P_{x}$ has less variance than the one with $P_{x}^{l}$. On the other hand, $P_{x}^{k}$ was significantly higher than $P_{x}^{l}$ in most of the coupling levels. Despite this, the nonlinear quantification was, in general, not significant according to the analysis of surrogates. This result suggests that $P_{x}^{k}$ might be over fitting the data.

**Figure 6:**
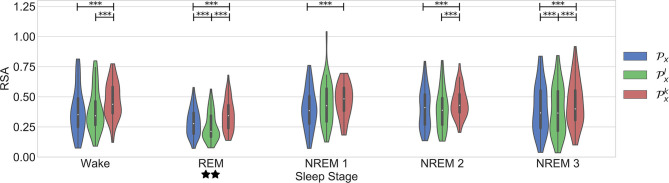
Results in the dataset of segments during different sleep stages. Significant differences between the parameters are marked with *. Significant differences between sleep stages are marked with ⋆.

The last evaluation aimed to analyze the change of the linear and nonlinear components of the cardiorespiratory quantifications during sleep stages. The results are in agreement with the findings presented in Bartsch et al. (2012). Figure 6 displays the results. The work presented in Penzel et al. (2016) shows that the regulation of the autonomic nervous system is different during each sleep stage. It is shown that the HR decreases during sleep, reaching a minimum during deep sleep, suggesting an increased parasympathetic activity. During REM sleep, mental activities are more active and thus a higher level of sympathetic activation is expected, resulting in a higher mean HR. In addition, the RSA is found significantly less active in REM sleep than in Non-REM sleep.

**Figure 7:**
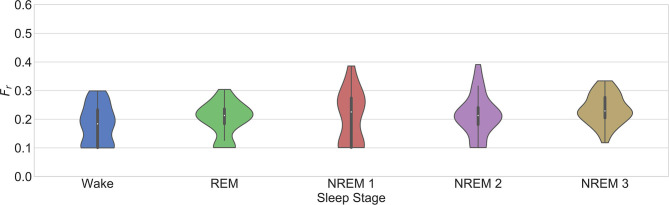
*F*_*r*_ for the selection of the model order in the third dataset as described in section 2.3.2.

This works confirms some aspects of these observations (see Figure 6). First, it is seen that RSA is significantly stronger during NREM compared to REM sleep. An interesting trend observed in the figure is a significantly stronger coupling during wake than in REM. This might have been due to the spectrum of the respiratory signals. In order to see the distribution of the respiratory patterns among the sleep stages, the frequency characteristic of the respiratory segments on each case were analyzed. Figure 7 shows the results. No significant differences were found. While the respiratory frequency has been shown to be an important confounder in cardiorespiratory analysis, this figure suggests that the characteristics of the respiratory patterns in the selected segments are similar and should not have a confounding effect. A second relevant observation is that the difference between the quantifications using $P_{x}^{l}$ and $P_{x}^{k}$ is smaller during deeper sleep stages. This result might suggest that the nonlinear influence of the respiration is more noticeable during lighter sleep stages in this dataset. However, it is important to highlight that the nonlinear quantification of the RSA with $P_{x}^{k}$ was not significantly different to its surrogates in most of the cases. Despite of this, as observed in the figure, the quantification with $P_{x}^{k}$ is significantly different to the part quantified by $P_{x}^{l}$ in all cases except NREM1. Finally, the significant differences between sleep stages are the same with all the parameters, with a significantly lower coupling only during REM.

The paper in Loula et al. (1994) suggested differences in the nonlinear part of the cardiorespiratory coupling during the application of anesthesia to healthy subjects. On the other hand, the paper in Chen et al. (2009) indicates that the nonlinear part of the cardiorespiratory coupling did not change significantly with different doses of propofol. Taking these works into account, the current paper tested the hypothesis that in the used dataset, the nonlinear part of the RSA might change according to the sleep stage. The results suggest that a nonlinear coupling component is not present in the interactions or that it might be too small to be captured using the proposed approach.

It is important to mention that this work has some limitations. First, the segments are extracted from OSA patients. The same study in healthy subjects might show different results. Second, the selection of the σ_*k*_ was found to be consistent. However, the tuning of the regularization term in the SVM problem is challenging. This is an open problem, not only for this application, and more research is required to investigate a more standard methodology to select this parameter.

## 4. Conclusions

In this work, a method to quantify the respiratory sinus arrhythmia based on regression models built with support vector machines, is presented. It allows to quantify the dominant form of coupling. The methods are a framework that will allow to analyze the nature of the regulatory mechanisms of the cardiorespiratory interactions in different conditions and diseases. The proposed approach was tested in simulated data. Taking into account the results obtained from the simulation, real data extracted from obstructive sleep apnea patients was analyzed. The results suggest that the nonlinear components of the RSA are not prominent during sleep stages and that the linear components are dominant in the analyzed datasets. The work in this paper is an application in which the evaluation of a physiological network provides insights of the functioning of the interactions between systems and demonstrates the added value of this framework. As a future work, the indexes described in this paper will be compared to other approaches such as linear and nonlinear calculations of transfer entropy.

## Data Availability Statement

The datasets presented in this article are not readily available because the raw data comes from patients. For this reason, it is subject to the European data-privacy policy regulations. Requests to access the datasets should be directed to carolina.varon@esat.kuleuven.be. The authors will try to provide an anonymized version of dataset in compliance with the privacy policy of the University Hospitals of Leuven, which is the owner of the data.

## Ethics Statement

The studies involving human participants were reviewed and approved by ethical committee of the university hospital UZ Leuven (S53746, S60319). The patients/participants provided their written informed consent to participate in this study.

## Author Contributions

JM and CV wrote the article and conducted the data analysis. SV and CV supervised the data analysis. PB, DT, and BB conducted the clinical trial and data collection. CV, SV, PB, DT, and BB reviewed and corrected the manuscript. All authors contributed to the article and approved the submitted version.

## Conflict of Interest

The authors declare that the research was conducted in the absence of any commercial or financial relationships that could be construed as a potential conflict of interest.

## Footnotes

^1^The implementation of the algorithms in Matlab® can be downloaded from: https://gitlab.esat.kuleuven.be/Biomed/RSA_NonLinear
